# Superior disrupting ferroelectric phase of (Ba Sr)Nb_2_O_6_ tungsten bronze structure via high entropy and cations disorder for ultrahigh breakdown strength

**DOI:** 10.1038/s41598-025-97510-5

**Published:** 2025-05-22

**Authors:** Marwa Kamal, Manal Mohammed Alhazmi, Abd El-razek Mahmoud

**Affiliations:** 1 Ferroelectric and Piezoelectric Lab, Physics Department, Faculty of Science, South Valley University, Qena, 83523 Egypt; 2 Physical sciences department, Faculty of Science, Jazan University, Jazan, Saudi Arabia

**Keywords:** Entropy, Breakdown strength, Interfacial polarization, Grain resistance, Activation energy, Energy science and technology, Materials science

## Abstract

High configurational entropy (ΔS) dielectric ceramics possess a unique competitive advantage in electronic applications due to their high-power density and excellent reliability. However, their development with superior energy storage density require high breakdown strength (E_b_) and suppression interfacial polarization (IFPs). Herein, high-entropy strategy disrupts ferroelectricity long-range ordering in tungsten bronze structure of (Ba, Sr)Nb_2_O_6_ ceramics via A/B-sites cation disorder. Hot pressing technique was performed to fabricate ((Ba_0.33−x_Nd_x_)Sr_0.33_Ca_0.33_))(Nb_2−x_Ti_x_)O_6_, (BSCNNT_X_) (x = 0.0, 0.05 and 0.1) in order to reduce the consumption energy during polyvinyl alcohol (PVA) burning. The obtained results reveal that ΔS increased from 1.09R for x = 0.0 to 1.49R for x = 0.1. The miss-matching between resistance of grain (R_g_) and grain boundary (R_gb_) completely suppressed at high ΔS resulting in pinched the interfacial polarization and oxygen vacancies effect. Superior elevation into activation energy (E_a_) was achieved at high ΔS where it increased from 0.041 eV at ΔS = 1.09R to 0.18 eV at ΔS = 1.49R attributed to superior weakly coupled of relaxor phase. This cascade effect results in outstanding breakdown strength ultimately achieving a E_b_ of 730 kV/cm in ((Ba_0.23_Nd_0.1_)Sr_0.33_Ca_0.33_))(Nb_1.9_Ti_0.1_)O_6_ sintered ceramics. This research presents an effective method for designing tetragonal tungsten bronze dielectric ceramics with ultra-high breakdown strength performance.

## Introduction

Due to increase the environmental protection concepts, the dielectric ceramic capacitors are consider a core components in several electronic applications of pulsed power devices such as energy vehicles, medical equipment, high energy weapon systems^1–3^. Comparison with batteries and super-capacitors, dielectric capacitors exhibit an ultra-high power density, ultra-fast charge/dis-charge rate, extended lifespan and excellent thermal and cycle stability^4,5^. Although, all these outstanding properties, to date the downsizing, miniaturization and integration requirements of pulse power electronics have been hard to achieved by dielectric capacitors due to their low recoverable energy storage density (W_rec_) and efficiency (η), dielectric breakdown strength (BDS) and low operation temperature range^6,7^. The large maximum polarization (P_max_), negligible residual polarization (P_r_) under high externally applied field (E_b_) are the essential parameters and key factors for optimizing the energy storage performance (ESPs) of dielectric capacitors^8,9^. To improve the ESPs of dielectric ceramics, several strategies have been utilized by researchers such as, reducing the grain size to sum-micro scale or mesoscopic scale^10,11^, domain engineering effect in a microscopic effect^12,13^, improvement polarization intensity and enhancement DBS by intrinsic and extrinsic means^14,15^. Recently, high entropy modulation is consider an alternative promising strategy and attract great attention to boost and augment energy storage capacity of perovskite dielectric capacitors ceramic. The configurational entropy (ΔS) of perovskite oxide systems can augmentable by increasing the numbers of random distribution cations on the same cite of lattice. High ΔS can induce high degree of relaxor behavior and increasing DBS value due to high disorder of atomic arrangement^16^. The configurational entropy of ABO_3_ perovskite structure can be calculated by the following Eq. 

1 $$\Delta S_{{Config}} = - R\left[ {\left( {\sum\nolimits_{{a = 1}}^{n} {x_{a} Lnx_{a} } } \right)_{{A{\text{ - }}site}} + \left( {\sum\nolimits_{{b = 1}}^{n} {x_{b} Lnx_{b} } } \right)_{{B{\text{ - }}site}} + 3\left( {\sum\nolimits_{{c = 1}}^{n} {x_{c} Lnx_{c} } } \right)_{{O{\text{ - }}site}} } \right]$$

Where x_a_, x_b_, and x_c_ are the mole fractions of cations presented at the A, B, and O sites, while R is the universal gas constant. The classification of perovskite materials based on configurational entropy can be categorized to three types. (i) high entropy materials where ΔS_config_ ≥ 1.5R, (ii) materials undergo medium entropy if ΔS_config_ is lie into range (1-1.5)R, and (iii) materials with low entropy if ΔS_config_ < 1R^17^.

In general, high entropy of dielectric ceramics can achieved highly degree of disordered polarization meticulously designed local structures. It’s well known that the cations disorder can induces lattice distortion which causes an impedes element diffusion through lattice crystal subsequently enhanced relaxor phase and energy storage capacity^18^. Therefor attributed to these desired properties of high entropy materials, high entropy strategy has been performed and applied to enhancement the ESPs of dielectric ceramics including materials with ABO_3_ structure^19^, materials with Bi-layer structure^20^, and materials with tungsten bronze structure^21^.

**Fig. 1:**
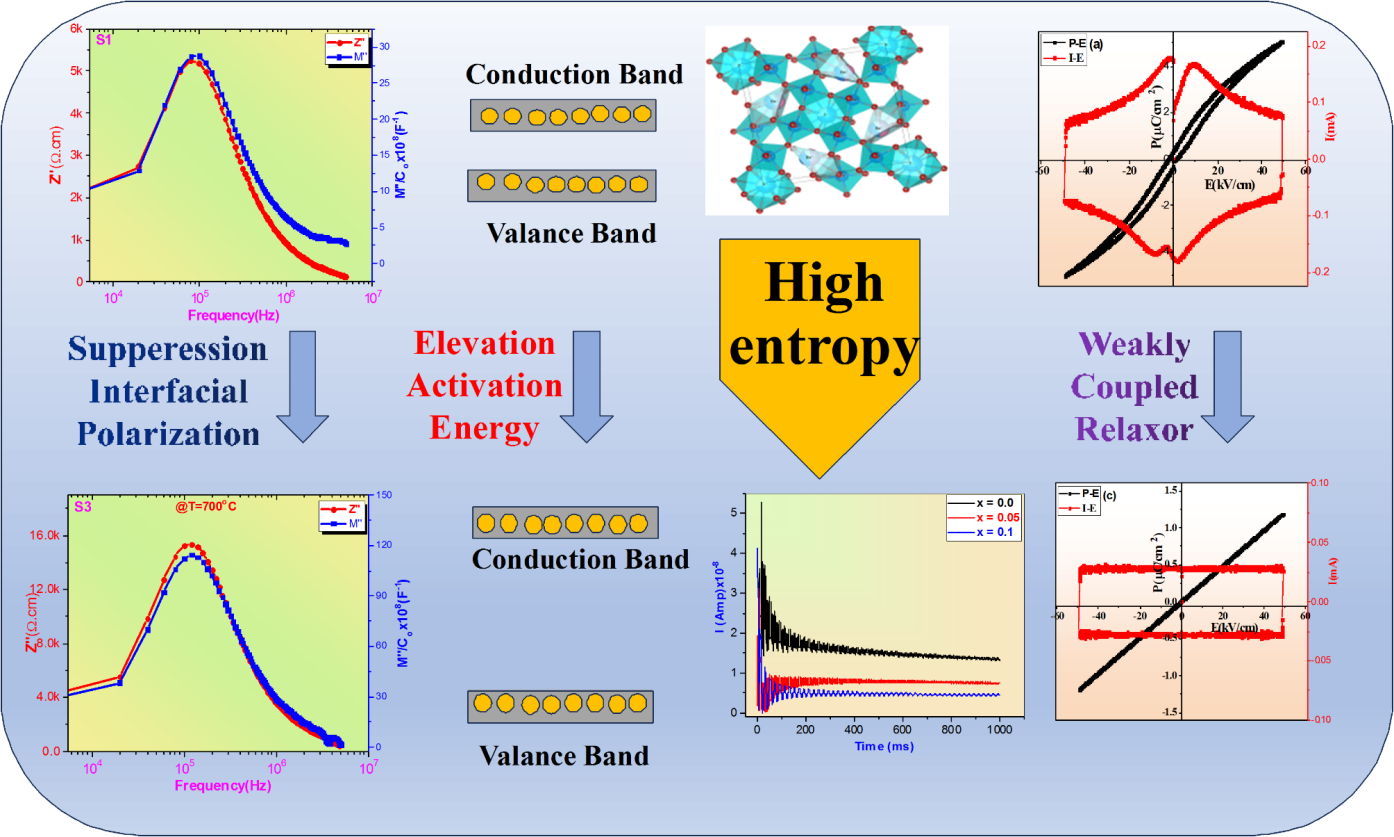
Schematic diagram of suppressing interfacial polarization via high-entropy, grain resistance and activation energy design for superior E_b_ and ESPs in BSN-based ceramics. The schematic diagram was generated using Powerpoint software 2019 available at website “https://www.microsoft.com/en-us/microsoft-365/download-office″.

The previous studies reported that the enhancement of entropy for ABO_3_ ceramics can induce ferroelectric-relaxor phase transition due to break the long-range order (LRO) of ferroelectricity and form polar nano-regions (PNRs). This phenomena can enhance the random field, suppress (Pr) and enhancement (η) of dielectric capacitors^22^. However, as the configurational entropy (S) increase, the cubic or pseudo-cubic phase become the main phase of perovskite crystal structure subsequently decreasing of P_max_ will take in place. So that, boost of breakdown strength (E_b_) is the only option for enhancement the ESPs of high entropy perovskite structure^23,24^. The suppression of interfacial polarization strategy is a powerful method for obtaining ultra-high E_b_, this strategy can induce random distribution of atoms which occurred by space charge and grain-grain boundary interface. Therefore, suppression of space charge is highly required for increasing E_b_ and enhancement ESPs of dielectric ceramics. The value of interfacial polarization or space charge effect can be occurred due to the difference between resistance of grains and resistance of grain boundaries. Generally, the resistance of grain boundary is much larger than the resistance of grain, therefore the enhancement of resistance of grains could be a heuristic for pinching the interfacial polarization subsequently enhanced E_b_. This can be achieved by highly insulation dielectric materials possess wide band gaps. Several studies and attempts have been paid by researchers for developments the entropy and enhancement ESPs of dielectric ceramics. For instance, Guo et al. reported high W_rec_ of 10 J/cm^3^ with moderate of η 89% in (Bi_0.2_Na_0.2_Sr_0.2_Ba_0.2_Ca_0.2_)TiO_3_ based Li_2_O_3_^25^. Changyuan Wang. et al. achieved W_rec_ of 11.8 J/cm^3^ and 86.4% of η at high Eb of 650 kV/cm in equimolar of BNT-based ceramics^26^. Furthermore, Yangfei Gao. et al. achieved high-temperature energy storage in Ba_0.4_Sr_0.3_Ca_0.3_Nb_1.7_Ta_0.3_O_6_ tungsten bronze-structured ceramics, where they obtained W_rec_ of 8.9 J/cm^3^ and η of 93% by high-entropy strategy and band gap engineering^27^. Engineering and increasing of band gap are essential factors for enhancement ESPs. Heighten the band gap means increasing the required energy for transfer the electrons from valence band to conduction band which can restrict the concentration of carriers charge into the conduction band subsequently reducing the conductivity and enhanced breakdown strength. This strategy of band gap is not only can optimizing the ESPs, but also it’s significant effect for achieving excellent thermal stability in a wide range of temperature^27^. Along with perovskite ceramics, the tungsten bronze structure (TBS) exhibit superior functional properties in dielectric, piezoelectric, pyroelectric, energy storage and electro-optic properties. The general formula of TBS can be expressed as (A1)2(A2)4(C)4(B1)2(B2)_8_O_30_, where monovalent cations such as K^1+^ and Na^1+^ or isovalent cations such as Ba^2+^, Sr^2+^, Ca^2+^, Pb^2+^ or trivalent rare earth cations such as La^3+^, Nd^3+^, Gd^3+^ can occupy A1 and A2 sites, while the B1 and B2 sites can be occupied by Nb^5+^, Ti^4+^, Zr^4+^, Sb^5+^ and the C-site can be taken by Li^1+^, Mg^2+^ where they possess very small ionic radius caompared to A1 and A2 sites. The TB can classified into unfilled, filled and fully-filled structure based on occupied of A and B-sites. If all A, B and C-sites are occupied, the TBS can refer to fully-filled structure, while if 1/6 of vacancies were presented into A-sites, the TBS can represent filled or unfilled structure. One of the important component as TBS is (Ba, Sr)Nb2O6 which exhibit tetragonal structure with unfilled structure due to presence 1/6 vacancies into A-sites which randomly between A1 and A2 into A-site. Both of Ba and Sr ions can distributed between A1 and An-sites based on the ionic radius. As ionic radius of Ba (1.64Å) is larger than the ionic radius of Sr (1.44Å) ions, so Ba ions will occupy A2 while Sr ions will occupy A1 and A2 site. In this case, the ferroelectric behavior is owing to the miss-matching between atoms in A2 site resulting in polarization effect by adjacent oxygen atoms as shown in Fig. 1. The ferroelectric relaxor phase transition can occurred when more than half of Sr can be set at A2-site. In the present work, both of Nd^3+^ and Ti^4+^ will occupied A and B-sites of (Ba, Ca, Sr)Nb_2_O_6_ (BCSN) to enhance their configurational entropy and suppression of interifical polarization via miss-matching resistance between grain and grain boundary strategy. On the other hand, this strategy is aim to enhance the breakdown strength and thermal stability of dielectric properties of BCSN ceramics by pinching polarity and weakly B-O bond. The weakly B-O bond at high entropy lead to enhanced relaxor degree of BCSNNT ceramics. Crystal structure, dielectric, ferroelectric and energy storage properties were studied and discussed in details.

## Experimental

### Fabrication of ceramic

In the present work, three different compositions of ((Ba_0.33−x_Nd_x_)Sr_0.33_Ca_0.33_))(Nb_2−x_Ti_x_)O_6_, (x = 0.0, 0.05 and 0.1) ceramics based on cation disorder into A and B-site of lattice were fabricated using hot pressing technique technique. The compositions were synthesized using high purity raw materials of BaCO_3_ (99.99% Sigma Aldrich), SrCO_3_ (99.99% Sigma Aldrich), Nd_2_O_3_ (99.99 Sigma Aldrich), CaCO_3_ (99.8% Sigma Aldrich), Nb_2_O_5_ (99.8% Sigma Aldrich), and TiO_2_ (99.99 Sigma Aldrich). After drying the powder at 200 °C to evaporate the humidity, the dried powder were weighted according to the stoichiometric proportions. All the powders were mixed together using zirconia ball and absolute ethanol. To obtain uniform mixing, the powder was subjected to ball milling for 24 h and 300 rpm. After milling time over, the powder was dried, sieved and calcined at 1100 °C for 2 h. The calciend powder was pressed using steel die between two hot plate at 200 °C for 30 min into disc with 10 mm in diameter and 1 mm thickness. Finally, the pressed samples were sintered in alumina crucible at sintering temperatures 1200 °C and 1300 °C for 2 h sintering time with 3 °C/min as heating cooling rate.

### Characterizations and measurements

The phase formation and crystal structure of all sintered ceramics were examined using X-ray diffraction (XRD with 0.15 nm of CuKα), while the surface morphology of non-polished samples were observed by scanning electron microscope (SEM). The electrical measurements were performed after polishing the samples, coated both surface by silver paste and annealing at 600 °C for 30 min to burn the electrode. The dielectric properties dependent temperature and frequency were carried out using computerized LCR meter. The ferroelectric properties including bi-polar, mono-polar P-E loop and current loop I-E were measured by Precision Premier II from Radiant Technologies, connected to a high voltage amplifier. For evaluation dielectric breakdown strength, the thickness of ten samples of each composition was reduced to 0.15 mm then coated with silver electrode.

## Results and discussion

**Fig. 2:**
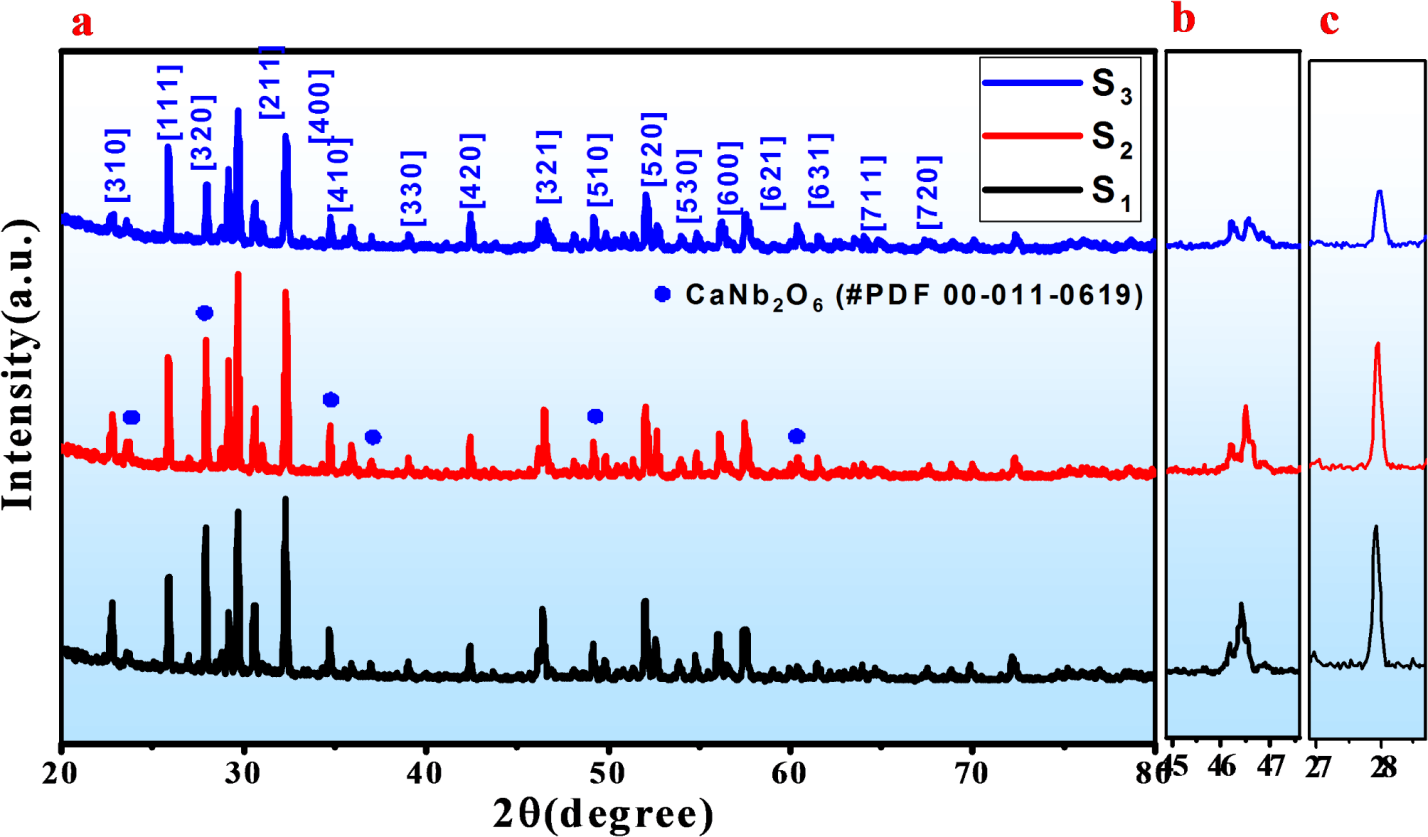
(**a**) X-ray diffraction patterns of (BSCNNTX) (x = 0.0, 0.05 and 0.1) )S1 = 0.0, S2 = 0.05 and S3 = 0.1 ceramics and (**b**) enlarge diffraction peak around 46° of 2-theta while (**c**) the enlarged main peak around 28 of 2-theta.

**Fig. 3:**
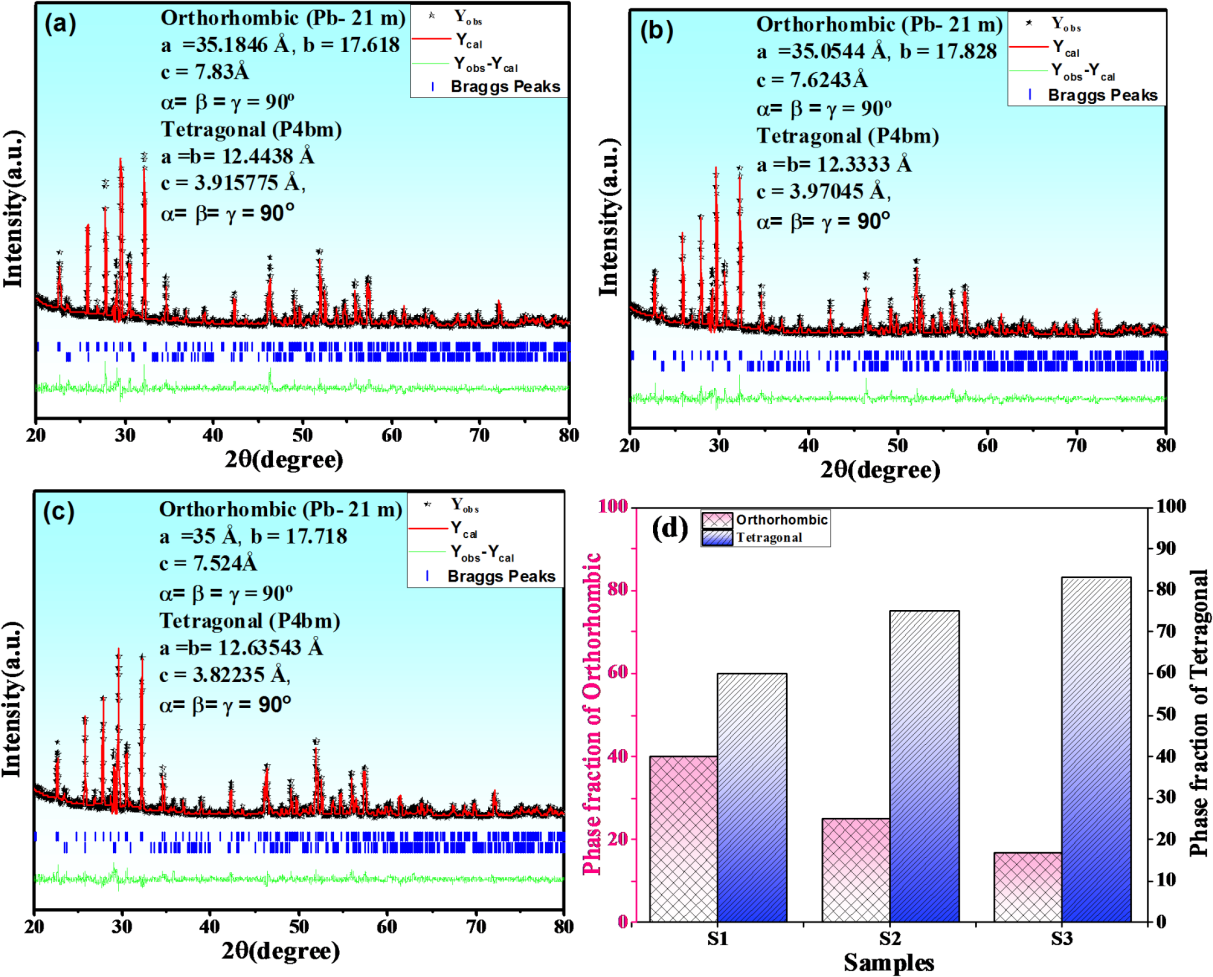
(**a**–**c**) Rietveld refinement pattern of (BSCNNTX) (x = 0.0 (**a**), x = 0.05 (**b**) and x = 0.1 (**c**) ceramics and (**d**) the volume fraction of O&T phases of each composition.

**Fig. 4:**
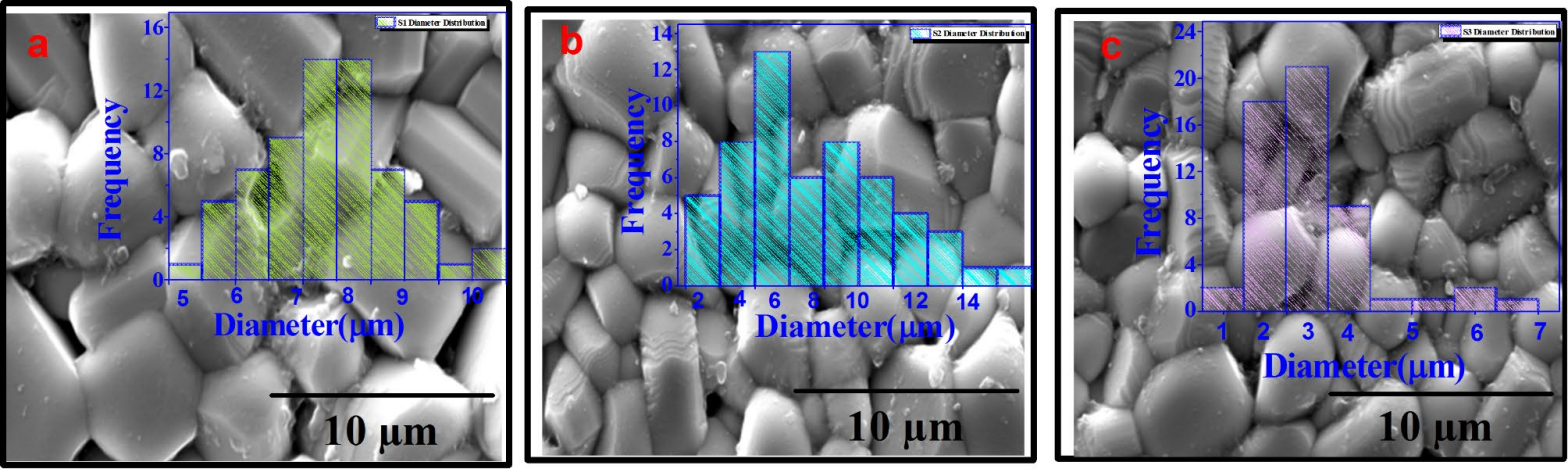
SEM microstructure and grain size distribution of ((Ba_0.33−x_Nd_x_)Sr_0.33_Ca_0.33_))(Nb_2−x_Ti_x_)O_6_, (x = 0.0, 0.05 and 0.1 represented by a, b and c respectively).

To determine the effect of configurational entropy on the crystal structure of (Ba, Ca, Sr)Nb_2_O_6_ tungsten bronze structure, XRD has been employed at ambient temperature for all present ceramics and the patterns were displayed into Fig. 2a. From the patterns, it is evident that all the samples possess tetragonal crystal structure which confirmed by present 2-peak arount 2θ = 46° Fig. 2b which signified the increasing in entropy is maintained the tetragonal structure phase. Mostly of patterns are belong to the crystal structure of (Ba_0.39_Sr_0.61_)Nb_2_O_6_ (PDF# 88-0785 and *P4bm* of space group) ceramic which denote both of Nd and Ti ions are successfully diffused into A and B-sites of BCSN to form BCSNNT solid solution An additional phase corresponding to an orthoromcic phase (O-phase) of CaNb_2_O_6_ (#PDF 00-011-0619, and *Pbcn* of space group) was detected. The present of impurity phase is attributed to the difficulty of Ca ions to occupy A2-sites and tend to occupy A1-sites due to its small ionic radius compared to Ba and Sr ions^28^. As it’s well known that, the forming of secondary phase is consider one of the strategies can be used for enhanced breakdown strength subsequently energy storage performance. The benefit of present secondary phase can be explained as when sintering composite ceramics, the second phase can play a “pinning effect”, thus significantly inhibiting grain growth. The reduced grain size and formed segregation phases are conducive to improve E_b_ according to the contraction relation ${E}_{b}\propto\:\:\frac{1}{G}$. In this case, one third will fill A1-sites while two-thirds will occupy A2-sites. Furthermore, as the ionic radius of Ti ions is smaller than ionic radius of Nb ions, therefore the B1-site will occupy by Ti^4+^ while B2 will occupy by Nb^5+^. it is clear to notice that via increasing the entropy, the volume fraction of T-phase increases and the crystal tend to be more symmetry. This reflect that the increasing in entropy resulting in increasing the degree of lattice symmetry which beneficial for decreasing the polarity, weakly B-O bond and enhanced relaxor phase. On the other hand, as the cations disorder increases, the patterns were observed shifted to higher diffraction angle reflect to increase the lattice shrinkage Fig. 2c. This attributed to the smaller ionic radius of (Nd^3+^ =1.27Å) compared to (Sr^2+^ = 1.44Å), (Ca^2+^ = 1.36Å) and (Ba^2+^ = 1.57Å) at A-site of lattice and smaller ionic radius of (Ti^4+^ = 0.60Å) compared to (Nb^5+^ = 0.69Å)^29^. Moreover in the absent addition of Nd and Ti ions, the mean ionic radius into A1 and A2-site is 1.36Å and 1.57Å respectively. Meanwhile by addition of Nd^3+^ into A-site, the mean ionic radius of A1-sites is 1.27Å and the mean ionic radius of A2-sites is 1.57Å, and by addition Ti4 + into B-site, the mean ionic radius of B1-site is 0.60Å and the mean ionic radius of B2-site is 0.69Å. By increasing the concentration of Nd and Ti ions into A and B-sites, the disparity in radius between A1, A2-sites and B1, B2-sites will increases which will affect on energy storage properties and breakdown strength^27^. On the other hand, the augmented difference between ionic radius at A and B-sites as entropy increases lead to reduction the grain size and increasing resistance of grain (R_g_). Rietveld refinement of XRD patterns were performed based on (T-phase, space group *P4bm*) using crystallographic information file (CIF) of (Ba_0.39_Sr_0.61_)Nb_2_O_6_ and (O-phase, space group *Pb−21 m*) using CIF of CaNb_2_O_6_ and the results were displayed into Fig. 3a–c with inset the lattice constant values and phase angle of each formed phase. The results shown that as entropy increased, the volume fraction of O-phase decreased while the volume fraction of T-phase increased indicating the system creep to high lattice symmetry. The volume fraction of each phase was calculated after achieving high reliability factors of R_wp_ and R_p_ < 2% and the results were displayed into Fig. 3d. The weakly polar phase (i.e. high volume fraction of T-phase) is conducive to enhancement E_b_ by reducing P_r_ value resulting in conducive to enhance comprehensive breakdown strength and ESP. Figure 4a–c displayed SEM micro-structure for surface morphology of all non polished ceramic samples. As clearly observed, near fully dense micro-structure along with lower grain size were achieved at high entropy sample while some pores exist into grain boundary were detected at low entropy. The present of voids and pores exist into grain boundary could be attributed to form the secondary phase which significantly induced inhibiting grain growth even at high sintering temperature. This evidence reflect that increase the grain resistance and suppressed interfacial polarization at high content of Nd and Ti ions. As entropy increase, the average grain size decreased as estimated using Image J software resulting in grain growth inhibitors which beneficial for enhancement breakdown strength and energy storage performance^30^.

**Fig. 5:**
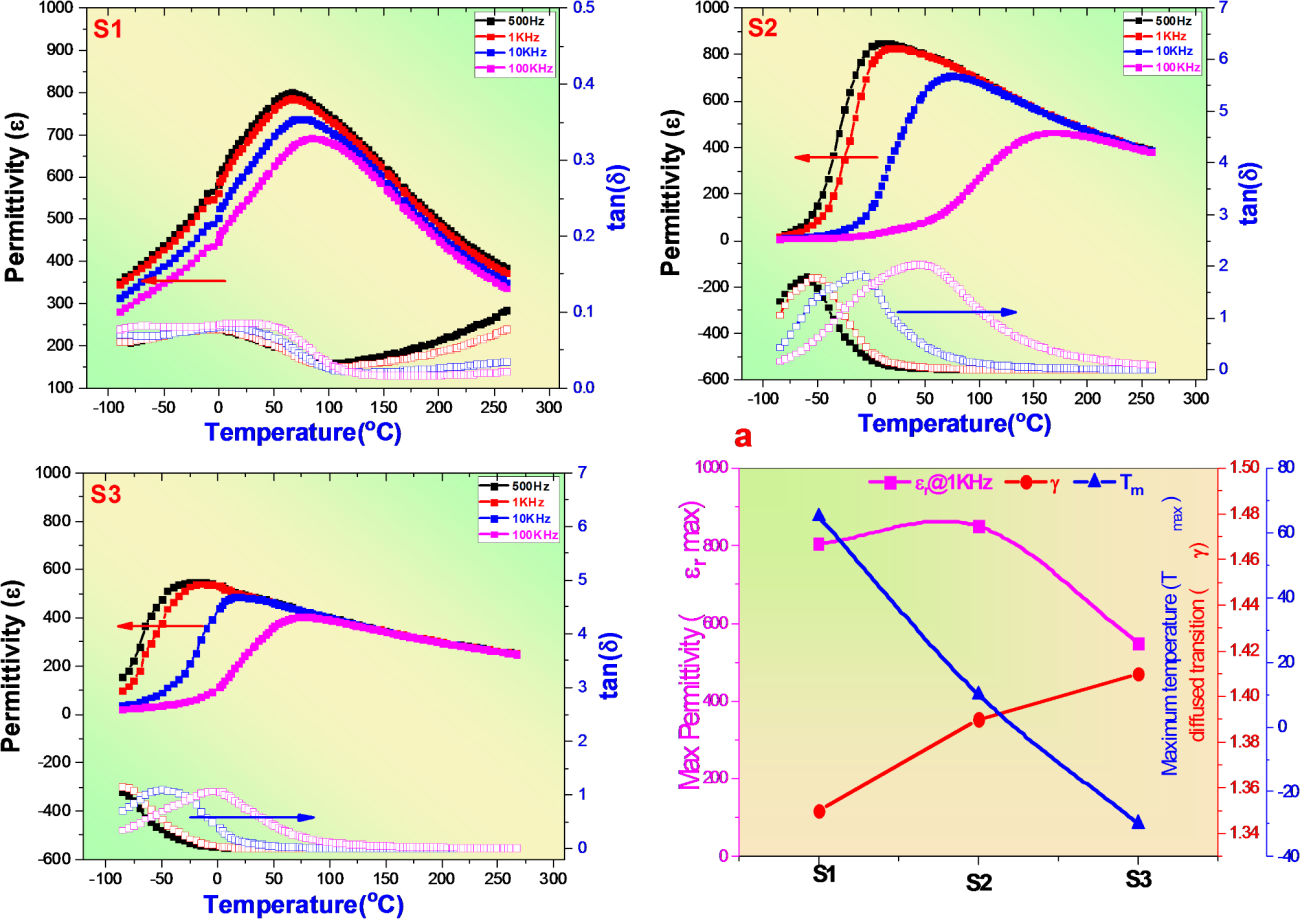
Permittivity dependent temperature at diff frequencies (0.5, 1, 10 and 100 kHz) of S1 (x = 0.0), S2 (x = 0.05) & S3 (x = 0.1), (**a**) represent ε_max_, T_m_ and γ of present ceramics.

The dielectric constant (ε_r_) and dielectric loss (tan δ) of all present ceramics were measured in a wide temperature range (−100 to 300 °C) and different frequencies (0.5, 1, 10 and 100 kHz) to distinguish the ferroelectric and relaxor phase and the results were depicted in Fig. 5. It’s worth to notice that pure BCSN with medium entropy (ΔS = 1.09R)^31^ shows only one less sharp permittivity peak along with less significant effect of frequency into dispersion behavior in the whole range of applied T. Furthermore a little shift in temperature corresponding to maximum permittivity (T_m_) to higher temperature has been observed as applied frequency increases. For instance, when the applied frequency increased from 0.5 to 100 kHz, the T_m_ increased from 65 to 85 °C. This evidence reflect present weakly degree of relaxor ferroelectric characteristic of BSCN ceramics^32^ or present non-ergodic relaxor phase. This behavior could be attributed to present Ca^2+^ ions into A2-site of lattice which can break the long-range order of BSN ferroelectricity and form BSCN relaxor behavior due to their small ionic radius compared to Sr and Ba ions. As entropy increases to 1.35R and 1.49R for x = 0.05 and 0.1 of Nd and Ti ions respectively, the dispersion of relaxor ferroelectric phase increased and high dispersion phase transition has been observed at highest entropy which reflect present ergodic relaxor phase. The T_m_ observed shifted to lower T along with decreasing maximum permittivity as entropy increases indicate enhanced the relaxor phase and weakly B–O bond^33^. Furthermore, the enehanced relaxation in the present ceramics may be due to the random distribution of Ti^4+^ and Nb^5+^ ions resulting in the occurrence of a local random field that favors the generation of randomly oriented PNRs^34^. This phenomenon is associated with the gradual switching of the relaxor process of the R-phase PNRs by the O-phase PNRs^35^. Also, worthily to notice that the maximum permittivity decreased from 805 to 390 when the T increased from (T_m_ = 65 °C) to T = 265 °C at pure BCSN sample while it was observed decreased only from 550 to 250 when the T increased from (T_m_ = −25 °C) to T = 270 °C. These reflect an excellent thermal stability into dielectric properties of the present samples as entropy increased from 1.09 to 1.49R Fig. 5a. This cascades influence are beneficial for enhanced breakdown strength, high thermal stability and improvement ESPs. Along with dielectric constant (ε_r_), the dielectric loss was measured at the same parameters of (ε_r_). The tan δ observed high at lower frequencies below phase transition and high at higher frequencies above the phase transition. This reflect to static polar nano-regions effect according to Depey’s relaxation theory below T_m_. Meanwhile the dynamic PNRs caused by the thermal activation of oxygen vacancies is dominated above T_m_^36,37^. The increasing in ΔS induced suppression of dielectric loss at high T which beneficial for enhanced energy storage efficiency.

To further investigate the ferroelectric and relaxor degree, the variation of permittivity as a function of T at 1 kHz as applied frequency at high T after Tc can be fitted with Curie-Weiss equation^38.^

2 $$\frac{1}{\epsilon\:}-\frac{1}{{\epsilon\:}_{m}}={\left(\frac{T-{T}_{m}}{C}\right)}^{\gamma}$$

**Fig. 6:**
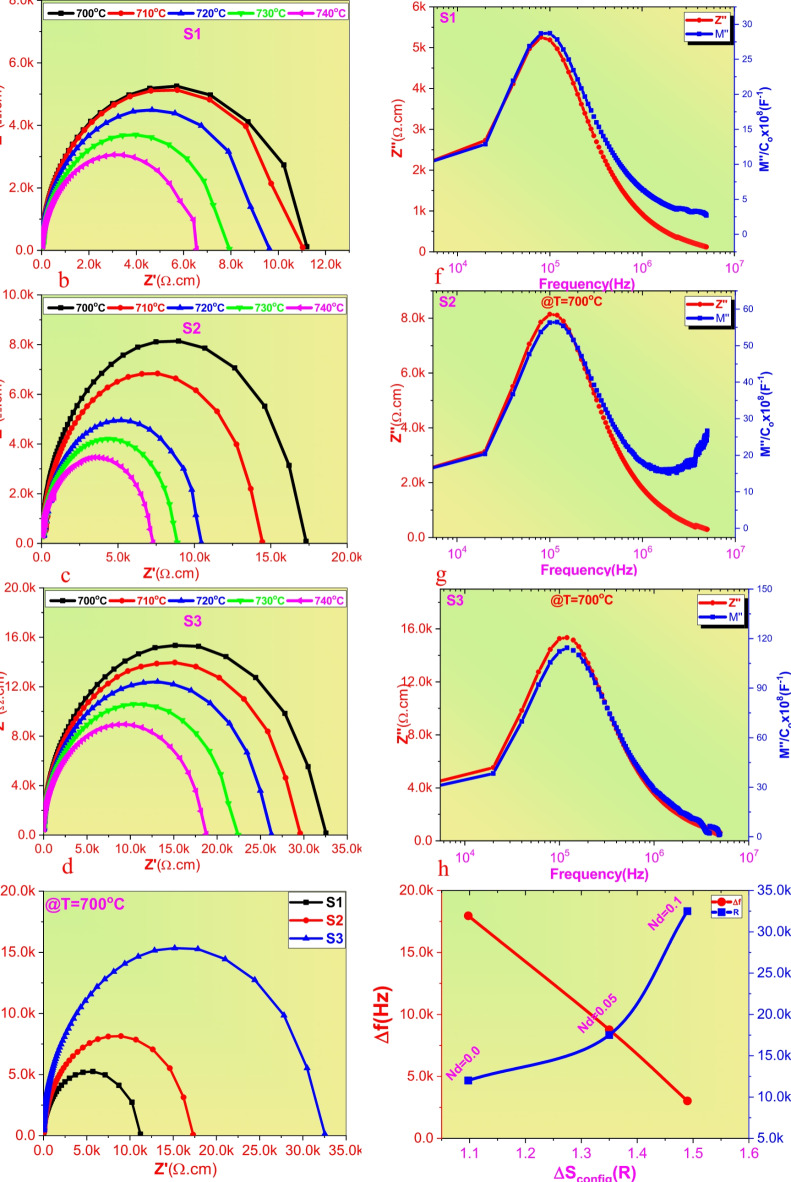
(**a**–**c**) are cole-cole plots of S1, S2 and S3 at different T (700–740 °C), (**d**) is comparison cole-cole plot of all samples at T = 700 ° C, (**e**–**g**) are variation of Z″ and M″ dependent frequency of present ceramics and (h) variation of Δf and cole radius dependent ΔS.

Where γ is describing the diffusion phase transition, C is a constant, ε is the dielectric constant at temperature T and ε_m_ is the maximum dielectric constant at T_m_. The γ value can reflect the ferroelectric degree. If the value equal to 1, the material undergo ideal ferroelectric, while the material can describe as ideal relaxor state if γ = 2. The fitting results displayed into Fig. 5a indicate that (ε−T) curve can undergo the modified Curie-Weiss law. The diffusion γ value of pure BCSN ceramic is 1.35 indicate present ferroelectric - relaxor coexistence phase, meanwhile 1.48 of γ was obtained at BCSN based 0.1 of Nd and Ti ions signified to enhanced the relaxor phase and thermal stability which beneficial for improvement ESPs characteristics. On the other hand, the interfacial polarization (IFPs) plays an important role for evaluation the energy storage performance of ceramics. This is because the main parameters related to ESPs such as grain size (G), optical band gap (E_b_), breakdown strength (BDS) and diffused phase transition (γ) are directly depend on IFPs values^39^. Previous studies reported that the IFPs can suppress by increasing configurational entropy^26,39^. Meanwhile the suppression of interface polarization can be occurred by increasing the resistance of grain (R_g_) up to pinched the difference between resistance of grain and resistance of grain boundary (R_gb_) (i.e (R_gb_−R_g_ ~ 0.0)). For achieving this purpose, we conducted to study both components of impedance Z (real impedance Zʹ and imagine impedance Z″ along with imagin part of modules M″ analysis. It’s well known that the total impedance can be described as Z = Zʹ−Z″, While the total modules M can be determined as M = Mʹ−jM″. The Neqyest plot of impedance (Zʹ & Z″) of all sintered ceramics were recorded at different temperatures (700, 710, 720, 730 and 740 °C) in frequency range (20Hz–5 MHz with 20000 Hz as frequency step) and the data were displayed into Fig. 6a–c. As clearly observed the cole-cole plots achieved semicircles for all sintered ceramics which signified both of grain and grain boundary have contribution to the graphs. It’s worth to notice that the the radius of impedance spectrum arc achieved largest value at highest entropy highlighting highest grain resistance and suppressed oxygen vacancies effect which beneficial for improvement breakdown strength and ESPs Fig. 6d. As it’s known that the variation of Z″ with frequency giving an information about resistance of grain boundary while M″ various frequency is reflect an information about resistance of grain^40^. Therefore to deduce how the resistance of R_g_ and R_gb_ changed with entropy, variation of Z″ and M″ as a function of frequency were calculated and the data depicted into Fig. 6e–g. All the samples shown present Z″ and M″ peaks indicate present R_g_ and R_gb_ effect. The interface polarization of ceramics is almost decreased as peaks overlapping between Z″ and M″ decreased where the values of R_g_ and R_gb_ will be close to each other. Several previous studies reported that the IFPs intensity can be calculated by the difference between R_g_ and R_gb_ activation energies or directly by the frequency gab (Δf) between the maximum of Z"and M″^26,39,41^. The variation of Δf as a function of entropy was displayed into Fig. 6h. Clearly observed that Δf exhibit lowest value at highest entropy which signified to the difference between R_g_ and R_gb_ completely suppression at high content of Nd and Ti ions. The present results strongly indicate that the high-entropy material can effectively reduce the interfacial polarization effect and synergistic simultaneously breakdown strength and energy storage performance.

**Fig. 7:**
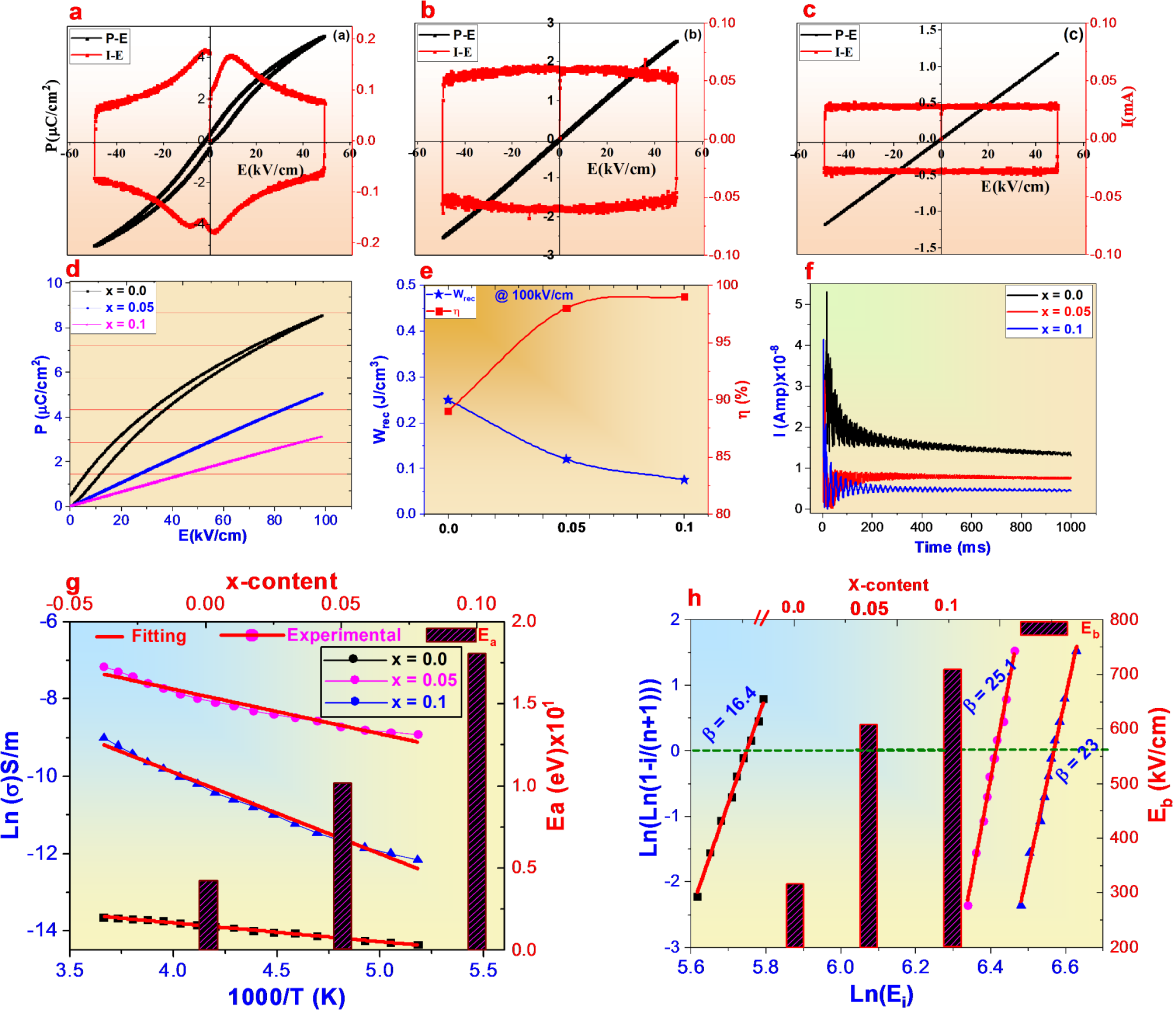
(**a**–**c**) are bi-polar P-I-E loops of (BSCNNT_X_) (x = 0.0 (**a**), 0.05 (**b**) and 0.1 (**c**) ceramics, (**d**) is uni-polar P-E loops @100 kV/cm, (**e**) is W_rec_ and η at 100 kV/cm, (**f**) is the leakage current of present ceramics at 50 kV/cm, (**g**) is conductivity dependent T along with Eg Vs x-content and (**h**) Weibull distribution analysis and breakdown strengths of presnt ceramics.

Figure 7a–c illustrate bi-polar polarization (P) and current (I) hysteresis loops dependent electric field E of (Ba_(0.33−x)_Nd_x_Sr_0.33_Ca_0.33_)(Nb_2−x_Ti_x_)O_6_ (x = 0.0, 0.05 and 0.1) at ambient temperature and 10 Hz waveform. Clearly observed the variation in entropy is clearly affect into ferroelectric properties. The sample with low entropy (x = 0.0), ΔS = 1.097R exhibit weakly relaxor phase with 5µC/cm^2^ of P_max_ and 0.35µC/cm^2^ of P_r_. As entropy ΔS increases to 1.35R at x = 0.05, both of P_max_ and P_r_ significantly decreases and linearity hysteresis P-E loops were obtained. This reflect enhanced weakly coupled polar nano-regions PNRs of the relaxor phase. The decreasing in P_max_ is signified to decrease the polarity and weakly B-O bond. The electric field is tends to be equivalent distributed between the grain and grain boundary phases when the interfacial polarization vanishes, (i.e. Δf→0) and this can be achieved at x = 0.1 where lowest P_max_ and P_r_ comprehensively were obtained^26,27^. This can be explained as, the imbalance of ions valance between donor Nb^5+^ and Ti^4+^ may force Ti^4+^ to become Ti^3+^ resulting in formation of Nb^5+^–Ti^3+^ ion pair. The electric field obtained by Nb^5+^–Ti^3+^ ion pair is benefited to obtain negligible remnant polarization and area loop into P-E curve. According to the constraint coupling between breakdown strength and P_max_, the elevation of E_b_ can be achieved by decreasing of P_max_ and (E_c_) comprehensively. Meanwhile, the current loop of x = 0.0 is characteristic by sharp current peak compared to other samples which is corresponding to the domain switching effect while I_max_ completely decayed with rectangular like-shape at high entropy samples. For investigation energy storage parameters of present ceramics as a function of entropy at low electric field, the uni-polar P-E loop were measured at 100 kV/cm and the data were displayed into Fig. 7d. The dramatically reduction in P_max_ reflect destruction of recoverable density (W_rec_), while the completely suppression of P_r_ at high ΔS resulting in superior achieving of energy storage efficiency (η). Based on uni-polar P-E loops, both of W_rec_ and η of present ceramics were calculated and deduced and the results were depicted in Fig. 7e. The decreasing in W_rec_ at highest ΔS is owing to decreasing the saturation polarization while the superior enhancement in η is attributed to negligible non-180 domain switching and high grain resistance resulting in linearity hysteresis loop where the charging and discharging curves are completely coincide to each others at x = 0.1 ceramics. To understand the reason for achieving superior efficiency at high entropy, the mechanism of electrical conduction in the material under investigation was studied by measure the leakage current at certain electric field. The leakage current can caused by one of the following or comprehensively effect of oxygen vacancies, space charge and interfacial polarization effect. The leakage current against time of the present ceramics was measured at 50 kV/cm and the results were displayed into Fig. 7f. As clearly observed, the increasing in entropy induced reducing the leakage current which denote suppression of conduction mechanism resulting in superior efficiency subsequently breakdown strength value at x = 0.1. One of the main parameters directly related to interfacial polarization and breakdown strength is activation energy (Ea) which can be calculated using the Arrhenius equation as the following.

3 $${\sigma\:}_{dc}={\sigma\:}_{o}{e}^{{-E}_{a/}{K}_{B}T}$$

where, ${\sigma\:}_{o}$is the pre-exponential factor, ‘Ea’ is the activation energy, K_B_’ is the Boltzmann constant and ‘T’ is the temperature (in K). The variation of Ln$\sigma\:$ dependent T for the present ceramics along with activation energy values were depicted into Fig. 7g. The results confirmed the Ea escalates from 0.041 eV at ΔS = 1.09R to 0.18 eV at ΔS = 1.49R. The increasing of activation energy denote a weakly coupling between polar clusters which making a challenging to form ordered polarized regions with high polarization^42^. This phenomenon is corresponding to delay the saturation polarization under high applied fields resulting in maintain a slimier P-E loop with neglection of P_r_ at x = 0.1 sample^43^.

For dielectric materials, breakdown strength (E_b_) is considered as an critical and very important crucial parameter for determining their energy storage performance. The Weibull distribution equation can be used to obtain the value of the breakdown strength. The value of E_b_ can be obtained from the following equations^30,44^

4 $${X}_{i}=Ln\:\left({E}_{i}\right)$$

5 $${Y}_{i}=Ln\left(Ln\left(\frac{1}{\left(1-\frac{i}{1+n}\right)}\right)\right)$$

X_i_ and Y_i_ are two variables of the Weibull expression. While, E_i_ presents the breakdown strength of samples (E_1_ < E_2_….< E_n_), i denotes the ordinal number of each sample where (i = 1, 2,…., n) and n is the total amount of samples (*n* = 10). The linear relationship between Xi and Yi can undergo the general formula ${Y}_{i}=\beta\:{X}_{i}\:+\:c$, where $\beta\:$ represent the reliability of the characteristic breakdown strength where as it’s value > 12 denote the high reliability of E_b_ value. While ${E}_{b}={e}^{{X}_{i}}$ when the value of corresponding Y_i_ is 0. Weibull data for the present ceramics along with E_b_ values are shown in Fig. 7h. In order to estimate the accurate E_b_ value of present ceramics, breakdown experiments were carried out on 10 samples of each component, and the standard Weibull distribution analysis was calculated according to the results. The data shown a linear relationship between X_i_ and Y_i_, which indicate all measurement data points are highly agreement with the Weibull distribution. The E_b_ can be extrapolated from the intersection of the fitted line and the X axis when Y_i_ = 0. It is reported that the breakdown strength is directly dependent on the grain size, band-gap, space charge^30,44,45^. The E_b_ increased from 310 kV/cm corresponding to ${\upbeta}$ = 16.4 at x = 0.0 to 730 kV/cm at x = 0.1 with high value of ${\upbeta}$ = 23.1. The superior E_b_ at high entropy is attributed to high grain resistance, high activation energy and low interfacial polarization which beneficial for enhancement weakly coupled relaxor phase degree.

## Conclusion

In summery, based on the pristine material of BSCN bronze structure, A/B-site were occupied by Nd and Ti ions respectively for enhanced their entropy and breakdown strength performance. By controlling entropy, high grain resistance and elevation activation energy a remarkable high breakdown strength E_b_ = 710 kV/cm was achieved in ((Ba_0.23_Nd_0.1_)Sr_0.33_Ca_0.33_))(Nb_1.9_Ti_0.1_)O_6_ sintered ceramics. Notably, the superior E_b_ is argued to be the reason that the high entropy and grain resistance can suppressed interfacial polarization and oxygen vacancies effect. Ferroelectric properties proves that high entropy lead to break ferroelectricity long range order resulting in highly dynamic and superior weakly coupled relaxor phase. The enhancement of E_b_ is responsible for the greatly enhanced energy storage performance in the BSCN ceramics, hold great potential for energy storage application across a broad temperature range.

## Data Availability

The datasets used and/or analysed during the current study available from the corresponding author on reasonable request.
